# Association of hand grip strength with rheumatoid arthritis and osteoarthritis in the Korean population: A large-scale cross-sectional study

**DOI:** 10.1371/journal.pone.0343150

**Published:** 2026-03-02

**Authors:** Jeong Hee Chi, Bum Ju Lee

**Affiliations:** 1 Department of Computer Science and Engineering, Konkuk University, Seoul, Republic of Korea; 2 Digital Health Research Division, Korea Institute of Oriental Medicine, Daejeon, Republic of Korea; Trinium Woman's Hospital, KOREA, REPUBLIC OF

## Abstract

**Background:**

Low hand grip strength (HGS) is associated with osteoarthritis (OA) and rheumatoid arthritis (RA), but no studies have simultaneously examined the associations of OA/RA with HGS indices. The objective was to examine the associations between absolute/relative HGS and the risk of OA, RA, and combined OA and RA (OR-arthritis).

**Methods:**

This large-scale cross-sectional study was based on data from the Korea National Health and Nutrition Examination Survey. A complex survey sample design was applied to the entire Korean population. Associations of OA, RA, and OR-arthritis with HGS and anthropometric indices were examined by binary logistic regression in unadjusted and adjusted analyses.

**Results:**

The prevalence of OA and RA was 8.2% and 1.1%, respectively, in men and 26.9% and 2.2%, respectively, in women. The prevalence of OR-arthritis was 1.4% in women. In men, OA was strongly associated with the waist-to-height ratio (WHtR) and HGS in both hands divided by the WHtR. RA had the strongest association with HGS in the dominant hand divided by waist circumference (WC) and with HGS in the dominant hand divided by WHtR. In women, OA showed a strong association with body mass index. RA was strongly associated with HGS in both hands and HGS in the dominant hand divided by height. Finally, OR-arthritis was strongly related to HGS in both hands divided by WC and HGS in both hands divided by weight.

**Discussion:**

The prevalence of OA was much greater than that of RA in both sexes. We found that the best index among anthropometric, absolute HGS, and relative HGS indices differed according to OA, RA, and OR-arthritis status and sex. The clinical implication of this study is that the association between RA and HGS was greater than that between OA and HGS in both sexes.

## Introduction

Osteoarthritis (OA) and rheumatoid arthritis (RA) are chronic progressive diseases associated with poor quality of life due to the destruction of joint function [1–5]. OA is more common in patients with arthritis and is a degenerative joint disease that occurs mainly in elderly individuals [4,6]. The potential risk factors for OA are obesity, aging, genetics, muscle weakness, joint laxity, and traumatic injury [1,3], and the symptoms of OA are inflammatory pain, stiffness, joint swelling, reduced function, and synovitis [2,7]. The pathological features of OA are cartilage destruction and degeneration, synovitis, and proliferation of subchondral bone [4,5], and anti-inflammatory drugs and physical exercise are used for the treatment of this disease [4]. RA is a chronic, heterogeneous, and systemic autoimmune disease in which immune cells release inflammatory cytokines and rheumatoid factors to activate joint inflammation [4,5]. RA occurs mainly in middle-aged individuals [8]. The pathological features of RA are pannus formation and chronic synovitis [5], and the symptoms are joint pain and morning stiffness [9]. Disease-modifying anti-rheumatic drugs are commonly used for the treatment of this disease [6,10].

Recently, many studies have suggested that patients with OA [11–17] or RA [18–20] have lower HGS values than healthy subjects in several countries. For example, the maximal HGS of patients with hand OA was 10% lower than that of healthy subjects [11]. RA prevalence was negatively associated with HGS in both men and women, and HGS was a strong predictor of health assessment and functional disability in patients with RA [18,19]. However, these studies on the association between HGS and RA examined only absolute dominant or nondominant HGS values, and no studies have examined associations between RA and relative HGS or between combined OA/RA (OR-arthritis) and HGS.

The objectives of this study were to examine the associations between absolute/relative HGS and types of arthritis (OA, RA, and OR-arthritis) and to determine the best HGS indices for identifying OA and RA among various absolute and relative HGS indices. We believe that identifying differences between OA/RA and HGS is important because OA and RA fundamentally differ in terms of pathogenesis, symptoms, prognosis, diagnosis, and treatment [4,5,8]. To our knowledge, this is the first study to report the associations of OA, RA, and OR-arthritis with relative and absolute HGS indices.

## Materials and methods

### Study population

**Fig 1:**
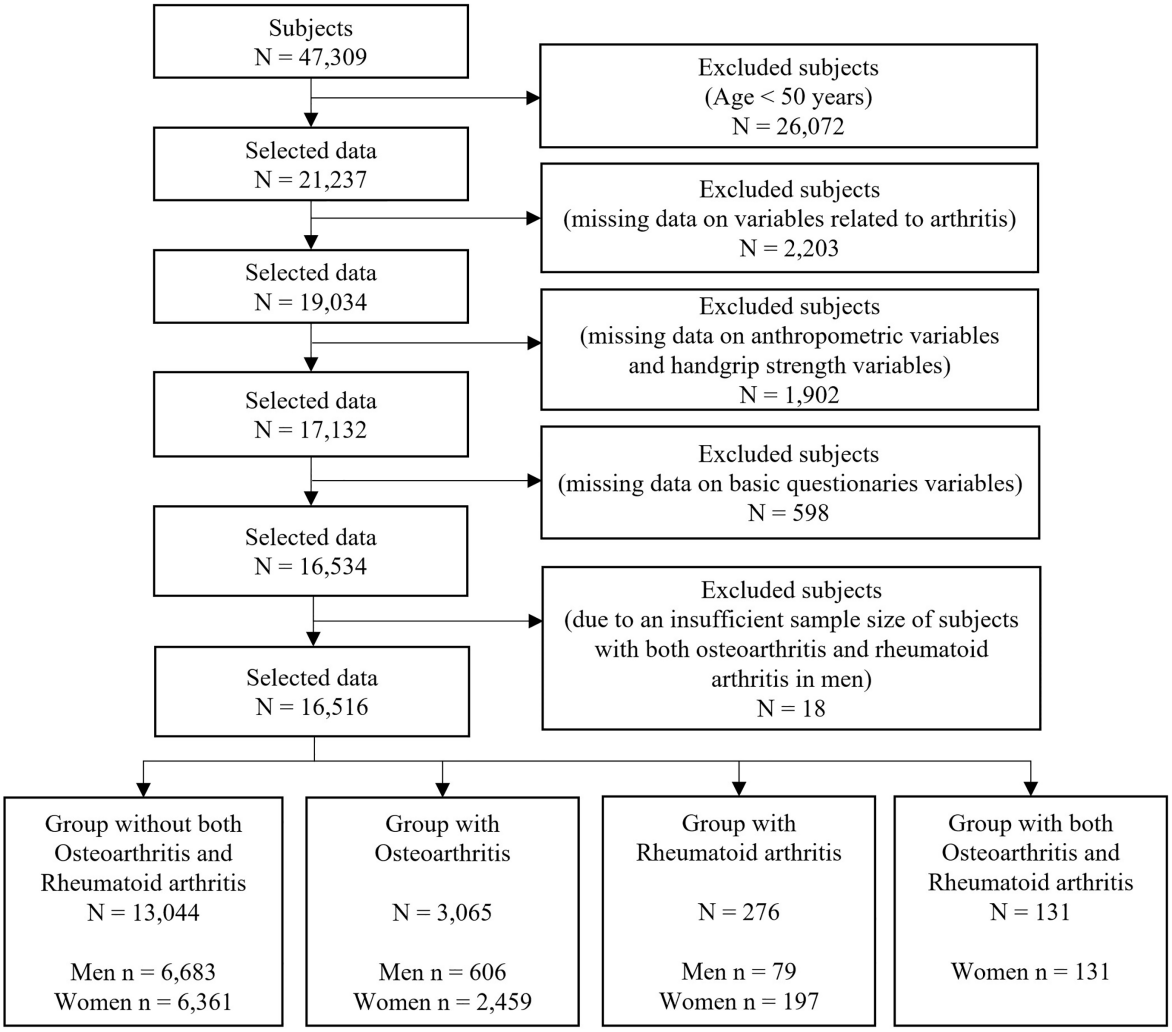
Sample selection procedure.

The present large-scale cross-sectional study was based on data from the Korea National Health and Nutrition Examination Survey (KNHANES) conducted by the Korea Disease Control and Prevention Agency (KDCA). The KNHANES data used to examine the health and nutritional status of the South Korean population included nationally representative and reliable statistics on biochemical and clinical profiles, physical examination data, socioeconomic status, health-related behavior data, and dietary intake data [21–24]. The KNHANES was carried out with the approval of the Institutional Review Board (IRB) of the KDCA (IRB: 2013-12EXP-03-5C, 2018-01-03-P-A, 2018-01-03-C-A) [22]. For the use of KNHANES data, we received approval from the Institutional Review Board of the Korea Institute of Oriental Medicine (IRB No. I-2209/009–001). All subjects participated in the survey gave their written informed consent. The authors did not have access to any personally identifiable data or information that would link the data to individuals’ identities. For the association of HGS with OA and RA, we used data from 2014 to 2019 because both HGS measurements and the major outcome variables were available only during this period; for example, data collected before 2014 did not include HGS measurements. For the purposes of this study, we accessed the data on 5 January 2024 (05/01/2024). The number of subjects included in this study was 47,309. In the step of the inclusion and exclusion of subjects, we selected subjects aged 50 years and over because there were very few subjects with OA and/or RA younger than 50 years old. We excluded subjects with missing data related to arthritis, HGS, anthropometry, or major sociodemographic characteristics. Additionally, we excluded male subjects with both OA and RA due to an insufficient sample size for analysis. Overall, a total of 16,516 subjects were included in the statistical analysis (13,044 subjects with neither OA nor RA, 3,065 subjects with OA, 276 subjects with RA, and 131 subjects with both OA and RA). More details on the inclusion and exclusion criteria of the subjects and procedures are shown in Fig. 1. The present study was performed in accordance with the principles of the Helsinki Declaration, and all methods were carried out in accordance with the guidelines of the KDCA [21,23,24].

### Definitions of osteoarthritis and rheumatoid arthritis

This was a retrospective cross-sectional study using data collected from the KNHANES. In KNHANES, OA was defined according to participants’ responses to questions during a face-to-face health interview: “Were you diagnosed with OA by a clinician or physician?” Patients who responded “Yes” were included in the OA group. RA was defined in the same way. Additionally, for patients with both OA and RA, those who answered “Yes” to the question “Have you been diagnosed with both OA and RA by a clinician or physician?” were included in the OR-arthritis group. Subjects who answered “No” to the question “Have you been diagnosed by a doctor as having OA or RA?”, were included in the nonarthritic group. To avoid respondent recall bias, the data were collected through a fact-to-face interview with experts or well-trained staff in compliance with strict guidelines [21,23,24].

### Covariates

The associations of all HGS indices with OA and RA were investigated before and after adjustment for covariates based on sociodemographic and health-related factors or characteristics obtained by an interview-based questionnaire. Based on previous literatures, we selected the following covariates: age [25–29], residential area (dichotomized as city or rural) [25,29], education level (consisting of elementary school or lower, middle school, high school, or university or higher) [17,26–29], employment status (dichotomized as no or yes) [25], household income quartile (consisting of low, middle-low, middle-high, or high) [28,29], alcohol consumption (dichotomized as yes or no) [17,28], smoking status (consisting of daily, former, or never) [17,26,28], and walking exercise [26,28]. In women, menopause (dichotomized as no or yes) was also examined as a potential effect modifier [30]. The detailed covariates are described in Table 1.

**Table 1:** Demographic characteristics of the subjects.

Variables	Men					Women						
	Nonarthritis	OA	p value^a^	RA	p value^b^	Nonarthritis	OA	p value^c^	RA	p value^d^	OR-arthritis	p value^e^
Number of subjects (%)	6,683	606 (8.2)		79 (1.1)		6,361	2,459 (26.9)		197 (2.2)		131 (1.4)	
Age (years)***	60.70 ± 0.14	66.05 ± 0.45	<0.001	62.37 ± 1.12	0.258	60.49 ± 0.15	67.37 ± 0.22	<0.001	63.92 ± 0.81	0.052	68.72 ± 0.73	<0.001
Residential areas			0.016		0.625			<0.001		0.596		0.575
City	80.50 (1.30)	75.80 (2.50)		82.30 (4.40)		82.10 (1.20)	76.70 (1.70)		82.20 (3.00)		82.76 (3.67)	
Rural	19.50 (1.30)	24.20 (2.50)		17.70 (4.40)		17.90 (1.20)	23.30 (1.70)		17.80 (3.00)		17.24 (3.67)	
Education***			<0.001		0.278			<0.001		0.314		<0.001
<= Elementary school	19.14 (0.61)	36.70 (2.40)		31.34 (6.19)		32.00 (0.80)	59.79 (1.21)		45.38 (3.88)		62.83 (5.06)	
Middle school	15.72 (0.57)	18.80 (1.90)		15.13 (4.53)		16.10 (0.60)	15.71 (0.84)		16.29 (2.86)		22.11 (4.37)	
High school	33.18 (0.72)	28.30 (2.20)		26.50 (6.01)		34.10 (0.80)	17.85 (0.91)		27.80 (3.87)		11.79 (2.91)	
>= University	31.96 (0.90)	16.20 (2.00)		27.03 (5.97)		17.80 (0.70)	6.66 (0.58)		10.53 (2.51)		3.28 (1.65)	
Employment status***			<0.001		0.281			<0.001		<0.001		0.002
No	27.14 (0.67)	43.90 (2.50)		34.70 (6.00)		50.60 (0.80)	62.40 (1.20)		71.10 (3.70)		68.30 (4.30)	
Yes	72.86 (0.67)	56.10 (2.50)		65.30 (6.00)		49.40 (0.80)	37.60 (1.20)		28.90 (3.70)		31.70 (4.30)	
Household income***			<0.001		0.910			<0.001		0.863		<0.001
Low	17.63 (0.59)	35.10 (2.30)		22.10 (5.30)		22.00 (0.70)	40.28 (1.23)		28.30 (3.60)		49.50 (4.60)	
Middle-low	24.70 (0.67)	28.30 (2.20)		22.30 (5.10)		25.40 (0.70)	25.59 (1.00)		24.20 (3.40)		22.10 (4.00)	
Middle-high	25.48 (0.69)	19.80 (2.00)		23.60 (5.60)		23.70 (0.70)	19.34 (0.96)		24.60 (3.80)		17.00 (3.50)	
High	32.19 (0.86)	16.80 (2.00)		32.00 (6.70)		28.80 (0.80)	14.79 (0.93)		22.90 (3.40)		11.30 (3.30)	
Alcohol consumption***			0.003		0.831			<0.001		0.038		<0.001
No	20.49 (0.60)	26.20 (2.00)		22.10 (5.40)		56.50 (0.80)	47.31 (1.24)		45.72 (3.91)		35.34 (4.69)	
Yes	79.51 (0.60)	73.80 (2.00)		77.90 (5.40)		43.50 (0.80)	52.69 (1.24)		54.28 (3.91)		64.66 (4.69)	
Smoking status***			<0.001		0.033			0.194		0.731		0.049
Daily	31.18 (0.73)	21.59 (2.05)		40.10 (6.70)		3.60 (0.30)	2.80 (0.40)		2.80 (1.30)		5.50 (2.20)	
Former	50.6 (0.73)	60.92 (2.34)		52.30 (6.70)		3.30 (0.20)	3.70 (0.40)		2.60 (1.00)		7.00 (2.50)	
Never	18.22 (0.56)	17.50 (1.73)		7.60 (2.80)		93.20 (0.40)	93.50 (0.60)		94.60 (1.70)		87.50 (3.30)	
Walking Exercise/week (min)***	103.91 ± 1.08	107.62 ± 3.47	0.308	103.82 ± 10.24	0.974	97.31 ± 1.06	100.14 ± 1.77	0.153	98.96 ± 5.31	0.862	98.53 ± 7.42	0.949
Menopause								<0.001		0.037		0.016
No	–	–	–	–	–	14.50 (0.60)	3.10 (0.40)		5.80 (2.20)		1.60 (1.60)	
Yes	–	–	–	–	–	85.50 (0.60)	96.90 (0.40)		94.20 (2.20)		98.40 (1.60)	
Blood pressure												
SBP (mmHg)	123.68 ± 0.24	125.53 ± 0.76	0.019	124.13 ± 1.66	0.856	122.24 ± 0.28	126.9 ± 0.41	<0.001	126.27 ± 1.52	0.069	126.07 ± 1.79	0.156
DBP (mmHg) ***	78.03 ± 0.16	75.25 ± 0.49	<0.001	77.25 ± 1.19	0.624	75.28 ± 0.15	74.34 ± 0.24	0.001	75.18 ± 1.01	0.873	72.67 ± 0.87	0.007
Anthropometrics												
Height (cm)***	168.33 ± 0.10	166.52 ± 0.30	<0.001	167.66 ± 0.88	0.540	155.43 ± 0.09	153.37 ± 0.14	<0.001	154.77 ± 0.48	0.803	153.39 ± 0.50	0.003
Weight (kg)***	68.80 ± 0.15	68.41 ± 0.45	0.414	68.24 ± 1.33	0.690	57.50 ± 0.13	59.31 ± 0.21	<0.001	56.3 ± 0.70	0.019	57.75 ± 0.74	0.815
BMI (kg/m2)*	24.24 ± 0.04	24.64 ± 0.14	0.007	24.24 ± 0.40	0.941	23.79 ± 0.05	25.18 ± 0.08	<0.001	23.48 ± 0.26	0.009	24.56 ± 0.32	0.174
WC (cm)***	86.91 ± 0.12	89.04 ± 0.42	<0.001	87.94 ± 1.12	0.434	80.86 ± 0.15	85.30 ± 0.22	<0.001	80.75 ± 0.65	0.053	84.76 ± 0.87	0.001
WHtR***	0.52 ± 0.00	0.54 ± 0.00	<0.001	0.53 ± 0.01	0.300	0.52 ± 0.00	0.56 ± 0.00	<0.001	0.52 ± 0.00	0.075	0.55 ± 0.01	<0.001
Dominant Hand*			0.363		0.201			0.575		0.017		0.901
Right	87.98 (0.50)	86.05 (1.66)		86.00 (50)		89.40 (0.40)	89.28 (0.76)		85.50 (2.70)		87.30 (3.10)	
Left	5.06 (0.32)	5.10 (0.98)		1.90 (1.50)		4.40 (0.30)	4.45 (0.45)		9.20 (2.20)		5.00 (1.90)	
Both	6.96 (0.40)	8.85 (1.35)		12.10 (4.80)		6.20 (0.40)	6.23 (0.62)		5.30 (1.70)		7.70 (2.70)	
Absolute HGS (kg)												
HGS-DH***	36.76 ± 0.12	33.8 ± 0.34	<0.001	33.69 ± 0.99	0.004	21.69 ± 0.08	20.05 ± 0.12	<0.001	19.23 ± 0.41	<0.001	19.32 ± 0.43	<0.001
HGS-BH***	39.05 ± 0.12	35.91 ± 0.35	<0.001	36.41 ± 1.13	0.034	23.49 ± 0.08	21.78 ± 0.12	<0.001	20.85 ± 0.42	<0.001	20.76 ± 0.44	<0.001
Relative HGS												
HGS-DH/HT (kg/Height) ***	0.22 ± 0.00	0.20 ± 0.00	<0.001	0.20 ± 0.01	0.003	0.14 ± 0.00	0.13 ± 0.00	<0.001	0.12 ± 0.00	<0.001	0.13 ± 0.00	<0.001
HGS-DH/WT (kg/Weight) ***	0.54 ± 0.00	0.50 ± 0.01	<0.001	0.50 ± 0.01	0.004	0.38 ± 0.00	0.34 ± 0.00	<0.001	0.34 ± 0.01	<0.001	0.34 ± 0.01	<0.001
HGS-DH/BMI (kg/BMI) ***	1.53 ± 0.00	1.38 ± 0.02	<0.001	1.40 ± 0.04	0.008	0.93 ± 0.00	0.81 ± 0.00	<0.001	0.83 ± 0.02	<0.001	0.80 ± 0.02	<0.001
HGS-DH/WC (kg/WC) ***	0.43 ± 0.00	0.38 ± 0.00	<0.001	0.39 ± 0.01	0.001	0.27 ± 0.00	0.24 ± 0.00	<0.001	0.24 ± 0.00	<0.001	0.23 ± 0.01	<0.001
HGS-DH/WHtR (kg/WHtR) ***	71.82 ± 0.26	0.20 ± 0.00	<0.001	64.8 ± 2.11	0.002	42.36 ± 0.20	36.57 ± 0.24	<0.001	37.21 ± 0.80	<0.001	35.42 ± 0.91	<0.001
HGS-BH/HT (kg/Height) ***	0.23 ± 0.00	0.22 ± 0.00	<0.001	0.22 ± 0.01	0.031	0.15 ± 0.00	0.14 ± 0.00	<0.001	0.13 ± 0.00	<0.001	0.13 ± 0.00	<0.001
HGS-BH/WT (kg/Weight) ***	0.57 ± 0.00	0.53 ± 0.00	<0.001	0.54 ± 0.01	0.026	0.41 ± 0.00	0.37 ± 0.00	<0.001	0.37 ± 0.01	<0.001	0.36 ± 0.01	<0.001
HGS-BH/BMI (kg/BMI) ***	1.62 ± 0.01	1.47 ± 0.02	<0.001	1.51 ± 0.05	0.036	1.00 ± 0.00	0.88 ± 0.01	<0.001	0.90 ± 0.02	<0.001	0.86 ± 0.02	<0.001
HGS-BH/WC (kg/WC) ***	0.45 ± 0.00	0.41 ± 0.00	<0.001	0.42 ± 0.01	0.009	0.29 ± 0.00	0.26 ± 0.00	<0.001	0.26 ± 0.01	<0.001	0.25 ± 0.01	<0.001
HGS-BH/WHtR (kg/WHtR) ***	76.28 ± 0.26	67.81 ± 0.77	<0.001	69.98 ± 2.33	0.015	45.86 ± 0.20	39.73 ± 0.26	<0.001	40.42 ± 0.90	<0.001	38.06 ± 0.95	<0.001

*:p < 0.05, **:p < 0.01, ***: p < 0.001. These values represent p values for sex differences between all men and women. Continuous data are represented as the mean ± standard error (SE). Categorical data are represented as percentages (SEs).

P values were obtained using Rao-Scott chi-squared tests for categorical variables and a general linear model for continuous variables. Comparisons show p value^a^ in non-arthritis vs. OA and p value^b^ in Non-arthritis vs. RA in men, and p value^c^ in non-arthritis vs. OA, p value^d^ in non-arthritis vs. RA, and p value^e^ in non-arthritis vs. OR-arthritis in women.

Abbreviations: HGS: handgrip strength, DH: dominant hand, BH: both hands, HGS-DH: maximum handgrip strength of the dominant hand, HGS-BH: maximum handgrip strength in both hands, HT: height, WT: weight, BMI: body mass index, WC: waist circumference, WHtR: waist-to-height ratio, OR: odds ratio, CI: confidence interval, OR-arthritis: both osteoarthritis and rheumatoid arthritis, OA: osteoarthritis, RA: rheumatoid arthritis, SBP: systolic blood pressure, DBP: diastolic blood pressure, HT: height, WT: weight, BMI: body mass index, WC: waist circumference, WHtR: waist-to-height ratio, OR: odds ratio, CI: confidence interval.

### Measurements

Anthropometric indices were measured by well-trained staff or experts according to standardized protocols. Weight and height were obtained by automatic measurement equipment in units of 0.1 kg and 0.1 cm, respectively (JENIX DS-102, Dong Sahn Jenix Co., Seoul, Korea). Waist circumference (WC) was measured to the nearest 0.1 cm using a flexible plastic tape (Seca 200, Hamburg, Germany). The waist-to-height ratio (WHtR) was calculated by dividing WC by height. HGS was assessed by well-trained health staff or experts using a digital grip strength dynamometer (T.K.K 5401, Japan; Takei Scientific Instruments Co., Ltd., Tokyo, Japan) with a standardized protocol. Participants who had a history of surgery on the hand or arm in the last three months or who had pain within the last seven days were excluded from the measurements. All subjects in the measurement step were asked to stand with their feet shoulder-width apart and keep their elbows and wrists from touching their body. HGS measurements were taken on the dominant hand and the other hand. Participants rested for approximately one minute after each measurement, and the measurement was repeated three times. The absolute HGS indices consisted of the maximum value in the dominant hand (HGS-DH) and the mean value of the maximum values in both hands (HGS-BH). Relative HGS indices were obtained by dividing each HGS-DH and HGS-BH by height, WHtR, WC, weight, and BMI. The detailed configuration of the HGS measurements is described in the literature [25,30].

### Statistical analysis

In the present study, all analyses were carried out using SPSS 28 (IBM SPSS, Inc., Chicago, IL, USA) in accordance with the guidelines for the use of raw data from the KNHANES, and a complex survey sample design was applied because the data were stratified, clustered, and weighted to represent the entire Korean population. The more detailed complex survey sample design used in this study is described in the literature [25,30]. For the statistical analysis of sex differences, we used Rao–Scott chi-square tests for categorical variables. Additionally, we used a t test with general linear models for continuous variables. Associations between OA/RA and HGS and anthropometric indices were examined using binary logistic regression after standardizing the data. Z-score standardization was applied because the units of the indices differed. Specifically, we examined the associations between the nonarthritic group and the OA group, between the nonarthritic group and the RA group, and between the nonarthritic group and the OR-arthritis group. We built three models according to the adjusted variables. Specifically, the crude model was not adjusted, Model 1 was adjusted for age, and Model 2 was adjusted for age, employment status, household income, residential area, education, smoking status, walking exercise, and alcohol consumption. In the interaction analysis between sex and HGS indices, the interaction effect was significant for all HGS indices (*P* < 0.005), except for HGS-DH, HGS-BH, HGS-DH/HT, and HGS-BH/HT in OA in the fully adjusted regression models. For the analysis of the data, we examined the multicollinearity between indices or variables using the variance inflation factor. Additionally, we assessed the linearity between the logit of the dependent variable and the independent variables by the Box-Tidwell test. Given the large sample size used in this study, we calculated Cohen’s *d* effect sizes for all HGS indices (*d* ≈ 0.3–0.5). OR and p values were calculated using complex sample binary logistic regression. Odds ratios are presented with 95% confidence intervals (CIs).

## Results

### Sociodemographic characteristics

Table 1 shows the sociodemographic characteristics of the variables used in the present study. The number of final participants in this study was 16,516, comprising 7,368 men (44.6%) and 9,148 women (55.4%). The prevalence of OA and RA was 8.2% and 1.1%, respectively, in men and 26.9% and 2.2%, respectively, in women. The prevalence of patients with both OA and RA was 1.4% among women. The prevalence of OA was much greater than that of RA in both men and women. The mean ages of the OA and RA groups were 66.05 ± 0.45 and 62.37 ± 1.12 years for men and 67.37 ± 0.22 and 63.92 ± 0.81 years for women, respectively. Additionally, the mean age of women with both OA and RA was 68.72 ± 0.73 years. Among the sociodemographic variables, age, education, employment status, household income, alcohol consumption, smoking status, walking exercise, and diastolic blood pressure (DBP) significantly differed between sexes. In men, all variables except for walking exercise were significantly different between nonarthritic patients and OA patients, and only smoking status significantly differed between nonarthritic patients and RA patients. In women, all variables except for smoking status and walking exercise were significantly different between nonarthritic patients and OA patients. A significant difference in employment status (p < 0.001), alcohol consumption (p = 0.038), and menopause status (p = 0.037) was detected between the nonarthritic and RA groups. All variables except for residential area, walking exercise, and SBP were significantly different between nonarthritic and OR-arthritis patients.

### Association of HGS with osteoarthritis and rheumatoid arthritis in men

Tables 2 and 3 show the associations of HGS with OA, RA, and OR-arthritis in men. In OA, the HGS-BH/WHtR showed the strongest association with OA according to the crude model (odds ratio (OR)=0.58 [0.53–0.64], p < 0.001], followed by the HGS-BH/WC (OR=0.59 [0.54–0.66], p < 0.001) and the HGS-DH/WHtR (OR=0.60 [0.54–0.66], p < 0.001). In age-adjusted Model 1, the WHtR (adj. OR=1.36 [1.22–1.51], adj. p < 0.001) and HGS-BH/WHtR (adj. OR=0.74 [0.65–0.84], adj. p < 0.001) was strongly associated with OA, and the associations persisted in Model 2 after adjusting for various covariates (WHtR: adj. OR=1.33 [1.20–1.47], adj. p < 0.001) and HGS-BH/WHtR: adj. OR=0.77 [0.68–0.87], adj. p < 0.001). In RA, HGS-DH/WC and HGS-DH/WHtR had the strongest associations with RA in all the crude models (HGS-DH/WC: OR=0.63 [0.49–0.81], p < 0.001; HGS-DH/WHtR: OR=0.64 [0.49–0.83], p = 0.001); Model 1 (HGS-DH/WC: adj. OR=0.62 [0.46–0.84], adj. p = 0.002 and HGS-DH/WHtR: adj. OR=0.62 [0.45–0.86], adj. p = 0.004), and Model 2 (HGS-DH/WC: adj. OR=0.62 [0.45–0.86], adj. p = 0.004 and HGS-DH/WHtR: adj. OR=0.63 [0.44–0.89], adj. p = 0.008).

**Table 2:** Associations of osteoarthritis (OA) with absolute HGS and relative HGS indices in men.

Variables	Crude		Model 1		Model 2	
	OR (95% CI)	p value	Adj. OR (95% CI)	Adj. p value	Adj. OR (95% CI)	Adj. p value
Anthropometrics						
Height	0.74 (0.67-0.82)	<0.001	0.89 (0.80-1.00)	0.042	0.94 (0.84-1.06)	0.332
Weight	0.96 (0.87-1.06)	0.410	1.17 (1.06-1.30)	0.002	1.21 (1.09-1.33)	<0.001
BMI	1.14 (1.04-1.26)	0.005	1.27 (1.16-1.40)	<0.001	1.27 (1.15-1.40)	<0.001
WC	1.29 (1.17-1.43)	<0.001	1.30 (1.17-1.44)	<0.001	1.30 (1.17-1.43)	<0.001
WHtR	1.45 (1.31-1.60)	<0.001	1.36 (1.22-1.51)	<0.001	1.33 (1.20-1.47)	<0.001
Absolute HGS						
HGS-DH	0.67 (0.61-0.73)	<0.001	0.89 (0.79-1.00)	0.042	0.91 (0.81-1.03)	0.124
HGS-BH	0.65 (0.59-0.72)	<0.001	0.87 (0.78-0.98)	0.026	0.91 (0.80-1.02)	0.112
Relative HGS						
HGS-DH/HT	0.69 (0.63-0.76)	<0.001	0.91 (0.81-1.02)	0.101	0.93 (0.83-1.04)	0.182
HGS-DH/WT	0.67 (0.61-0.74)	<0.001	0.81 (0.73-0.89)	<0.001	0.81 (0.73-0.90)	<0.001
HGS-DH/BMI	0.62 (0.56-0.69)	<0.001	0.77 (0.69-0.86)	<0.001	0.79 (0.70-0.88)	<0.001
HGS-DH/WC	0.61 (0.55-0.68)	<0.001	0.77 (0.69-0.87)	<0.001	0.79 (0.71-0.89)	<0.001
HGS-DH/WHtR	0.60 (0.54-0.66)	<0.001	0.76 (0.67-0.86)	<0.001	0.79 (0.70-0.89)	<0.001
HGS-BH/HT	0.67 (0.61-0.74)	<0.001	0.90 (0.80-1.01)	0.069	0.92 (0.82-1.04)	0.163
HGS-BH/WT	0.65 (0.59-0.72)	<0.001	0.79 (0.71-0.88)	<0.001	0.80 (0.72-0.88)	<0.001
HGS-BH/BMI	0.60 (0.55-0.67)	<0.001	0.75 (0.67-0.84)	<0.001	0.78 (0.69-0.87)	<0.001
HGS-BH/WC	0.59 (0.54-0.66)	<0.001	0.75 (0.67-0.85)	<0.001	0.78 (0.69-0.88)	<0.001
HGS-BH/WHtR	0.58 (0.53-0.64)	<0.001	0.74 (0.65-0.84)	<0.001	0.77 (0.68-0.87)	<0.001

OR and p values were obtained from the crude and adjusted analyses using complex sample binary logistic regression. Odds ratios were estimated with 95% confidence intervals.

Model 1. Adjusted for age.

Model 2. Adjusted for age, residential area, education, employment status, household income, alcohol consumption, smoking status, and walking exercise.

Abbreviations: HGS: handgrip strength, DH: dominant hand, BH: both hands, HGS-DH: maximum handgrip strength of the dominant hand, HGS-BH: maximum handgrip strength in both hands, HT: height, WT: weight, BMI: body mass index, WC: waist circumference, WHtR: waist-to-height ratio, OR: odds ratio, CI: confidence interval.

**Table 3:** Associations of rheumatoid arthritis (RA) with absolute HGS and relative HGS indices in men.

Variables	Crude		Model 1		Model 2	
	OR (95% CI)	p value	Adj. OR (95% CI)	Adj. p value	Adj. OR (95% CI)	Adj. p value
Anthropometrics						
Height	0.89 (0.67-1.19)	0.443	0.95 (0.69-1.30)	0.734	0.98 (0.71-1.35)	0.895
Weight	0.94 (0.72-1.24)	0.678	1.00 (0.76-1.32)	0.995	1.04 (0.77-1.39)	0.802
BMI	1.00 (0.76-1.31)	1.000	1.03 (0.79-1.35)	0.809	1.06 (0.80-1.40)	0.700
WC	1.14 (0.87-1.48)	0.353	1.14 (0.87-1.48)	0.337	1.15 (0.87-1.52)	0.324
WHtR	1.20 (0.90-1.58)	0.209	1.17 (0.88-1.56)	0.281	1.17 (0.87-1.56)	0.292
Absolute HGS						
HGS-DH	0.66 (0.51-0.85)	0.001	0.65 (0.48-0.87)	0.005	0.66 (0.47-0.91)	0.012
HGS-BH	0.70 (0.52-0.94)	0.016	0.70 (0.48-1.01)	0.054	0.71 (0.48-1.06)	0.095
Relative HGS						
HGS-DH/HT	0.66 (0.52-0.84)	0.001	0.65 (0.49-0.86)	0.003	0.66 (0.48-0.89)	0.008
HGS-DH/WT	0.67 (0.52-0.86)	0.002	0.68 (0.52-0.90)	0.006	0.68 (0.51-0.90)	0.008
HGS-DH/BMI	0.66 (0.51-0.87)	0.003	0.67 (0.49-0.92)	0.012	0.67 (0.49-0.93)	0.017
HGS-DH/WC	0.63 (0.49-0.81)	<0.001	0.62 (0.46-0.84)	0.002	0.62 (0.45-0.86)	0.004
HGS-DH/WHtR	0.64 (0.49-0.83)	0.001	0.62 (0.45-0.86)	0.004	0.63 (0.44-0.89)	0.008
HGS-BH/HT	0.70 (0.52-0.93)	0.014	0.70 (0.49-0.99)	0.045	0.71 (0.49-1.04)	0.076
HGS-BH/WT	0.71 (0.54-0.93)	0.012	0.73 (0.54-0.98)	0.036	0.73 (0.53-0.99)	0.043
HGS-BH/BMI	0.70 (0.52-0.94)	0.018	0.72 (0.51-1.01)	0.057	0.72 (0.50-1.04)	0.076
HGS-BH/WC	0.66 (0.50-0.88)	0.004	0.66 (0.47-0.93)	0.017	0.67 (0.47-0.96)	0.028
HGS-BH/WHtR	0.67 (0.50-0.90)	0.007	0.66 (0.45-0.96)	0.030	0.67 (0.46-1.00)	0.049

OR and p values were obtained from the crude and adjusted analyses using complex sample binary logistic regression. Odds ratios were estimated with 95% confidence intervals.

Model 1. Adjusted for age.

Model 2. Adjusted for age, residential area, education, employment status, household income, alcohol consumption, smoking status, and walking exercise.

Abbreviations: HGS: handgrip strength, DH: dominant hand, BH: both hands, HGS-DH: maximum handgrip strength of the dominant hand, HGS-BH: maximum handgrip strength in both hands, HT: height, WT: weight, BMI: body mass index, WC: waist circumference, WHtR: waist-to-height ratio, OR: odds ratio, CI: confidence interval.

### Associations of HGS with osteoarthritis, rheumatoid arthritis, and OR-arthritis in women

Tables 4–6 present the associations of HGS with OA, RA, and OR-arthritis in women. In OA, HGS-DH/BMI, HGS-DH/WHtR, HGS-BH/BMI, HGS-BH/WC, and HGS-BH/WHtR had similarly greater associations with OA than did the other indices in the crude model. However, BMI showed the strongest association with OA in Model 1 (adj. OR=1.51 [1.43–1.60], adj. p < 0.001) and Model 2 (adj. OR=1.47 [1.39–1.55], adj. p < 0.001), followed by WC and WHtR. In RA, HGS-DH, HGS-BH, HGS-DH/HT, and HGS-DH/HT had similar associations with RA compared to other indices in the crude model, but in the adjusted models, RA showed the strongest association with HGS-BH and HGS-DH/HT in Model 1 (adj. OR=0.64 [0.53–0.77], adj. p < 0.001; adj. OR=0.63 [0.53–0.75], adj. p < 0.001) and Model 2 (adj. OR=0.63 [0.52–0.77], adj. p < 0.001; adj. OR=0.63 [0.52–0.75], adj. p < 0.001). In the OR-arthritis group (patients with both OA and RA), OR-arthritis was strongly associated with HGS-BH/WC (OR=0.50 [0.42–0.60], p < 0.001) and HGS-BH/WHtR (OR=0.50 [0.42–0.59], p < 0.001) in the crude model. In Model 1, WC (adj. OR=1.32 [1.10–1.59], adj. p = 0.003) and HGS-BH/WC (adj. OR=0.74 [0.60–0.91], adj. p = 0.004) had a slightly greater association with OR-arthritis than did the other indices. In Model 2, OR-arthritis was highly associated with HGS indices such as HGS-BH/WC (adj. OR=0.75 [0.61–0.93], adj. p = 0.009) and HGS-BH/WT (adj. OR=0.77 [0.64–0.93], adj. p = 0.007) compared to the other indices.

**Table 4:** Associations of osteoarthritis (OA) with absolute HGS and relative HGS indices in women.

Variables	Crude		Model 1		Model 2	
	OR (95% CI)	p value	Adj. OR (95% CI)	Adj. p value	Adj. OR (95% CI)	Adj. p value
Anthropometrics						
Height	0.71 (0.67-0.75)	<0.001	1.00 (0.93-1.06)	0.897	1.05 (0.98-1.12)	0.152
Weight	1.23 (1.16-1.29)	<0.001	1.46 (1.38-1.55)	<0.001	1.44 (1.37-1.53)	<0.001
BMI	1.51 (1.43-1.60)	<0.001	1.51 (1.43-1.60)	<0.001	1.47 (1.39-1.55)	<0.001
WC	1.64 (1.55-1.73)	<0.001	1.46 (1.37-1.54)	<0.001	1.41 (1.33-1.49)	<0.001
WHtR	1.79 (1.69-1.90)	<0.001	1.47 (1.38-1.56)	<0.001	1.41 (1.32-1.50)	<0.001
Absolute HGS						
HGS-DH	0.71 (0.67-0.75)	<0.001	1.01 (0.95-1.07)	0.817	1.01 (0.95-1.07)	0.795
HGS-BH	0.70 (0.67-0.74)	<0.001	1.02 (0.96-1.08)	0.547	1.02 (0.96-1.09)	0.555
Relative HGS						
HGS-DH/HT	0.74 (0.70-0.78)	<0.001	1.01 (0.95-1.08)	0.661	1.01 (0.95-1.07)	0.870
HGS-DH/WT	0.63 (0.60-0.66)	<0.001	0.78 (0.73-0.83)	<0.001	0.79 (0.74-0.83)	<0.001
HGS-DH/BMI	0.58 (0.55-0.62)	<0.001	0.77 (0.72-0.82)	<0.001	0.78 (0.73-0.83)	<0.001
HGS-DH/WC	0.59 (0.56-0.63)	<0.001	0.82 (0.76-0.87)	<0.001	0.83 (0.78-0.89)	<0.001
HGS-DH/WHtR	0.58 (0.54-0.61)	<0.001	0.81 (0.76-0.87)	<0.001	0.83 (0.78-0.89)	<0.001
HGS-BH/HT	0.74 (0.70-0.78)	<0.001	1.02 (0.97-1.09)	0.419	1.01 (0.96-1.08)	0.644
HGS-BH/WT	0.62 (0.58-0.65)	<0.001	0.77 (0.73-0.82)	<0.001	0.78 (0.73-0.83)	<0.001
HGS-BH/BMI	0.57 (0.54-0.61)	<0.001	0.76 (0.71-0.81)	<0.001	0.78 (0.73-0.83)	<0.001
HGS-BH/WC	0.58 (0.55-0.62)	<0.001	0.81 (0.76-0.86)	<0.001	0.83 (0.78-0.88)	<0.001
HGS-BH/WHtR	0.57 (0.54-0.60)	<0.001	0.81 (0.76-0.86)	<0.001	0.83 (0.78-0.89)	<0.001

OR and p values were obtained from the crude and adjusted analyses using complex sample binary logistic regression. Odds ratios were estimated with 95% confidence intervals.

Model 1. Adjusted for age.

Model 2. Adjusted for age, residential area, education, employment status, household income, alcohol consumption, smoking status, walking exercise and menopause.

Abbreviations: HGS: handgrip strength, DH: dominant hand, BH: both hands, HGS-DH: maximum handgrip strength of the dominant hand, HGS-BH: maximum handgrip strength in both hands, HT: height, WT: weight, BMI: body mass index, WC: waist circumference, WHtR: waist-to-height ratio, OR: odds ratio, CI: confidence interval.

**Table 5:** Associations of rheumatoid arthritis (RA) with absolute HGS and relative HGS indices in women.

Variables	Crude		Model 1		Model 2	
	OR (95% CI)	p value	Adj. OR (95% CI)	Adj. p value	Adj. OR (95% CI)	Adj. p value
Anthropometrics						
Height	0.89 (0.76-1.05)	0.172	1.08 (0.90-1.30)	0.423	1.10 (0.90-1.34)	0.344
Weight	0.86 (0.72-1.03)	0.106	0.94 (0.79-1.11)	0.452	0.93 (0.78-1.10)	0.392
BMI	0.90 (0.76-1.07)	0.246	0.89 (0.75-1.06)	0.207	0.88 (0.73-1.05)	0.144
WC	0.99 (0.85-1.15)	0.879	0.92 (0.78-1.08)	0.309	0.90 (0.76-1.06)	0.192
WHtR	1.02 (0.88-1.19)	0.754	0.89 (0.75-1.06)	0.197	0.86 (0.72-1.03)	0.103
Absolute HGS						
HGS-DH	0.61 (0.52-0.72)	<0.001	0.66 (0.55-0.79)	<0.001	0.66 (0.54-0.80)	<0.001
HGS-BH	0.60 (0.51-0.70)	<0.001	0.64 (0.53-0.77)	<0.001	0.63 (0.52-0.77)	<0.001
Relative HGS						
HGS-DH/HT	0.61 (0.52-0.71)	<0.001	0.65 (0.55-0.78)	<0.001	0.65 (0.54-0.78)	<0.001
HGS-DH/WT	0.65 (0.57-0.75)	<0.001	0.71 (0.61-0.82)	<0.001	0.71 (0.61-0.83)	<0.001
HGS-DH/BMI	0.65 (0.56-0.76)	<0.001	0.72 (0.61-0.85)	<0.001	0.72 (0.61-0.86)	<0.001
HGS-DH/WC	0.62 (0.54-0.72)	<0.001	0.67 (0.57-0.80)	<0.001	0.68 (0.57-0.82)	<0.001
HGS-DH/WHtR	0.63 (0.54-0.73)	<0.001	0.68 (0.57-0.82)	<0.001	0.69 (0.57-0.83)	<0.001
HGS-BH/HT	0.59 (0.50-0.69)	<0.001	0.63 (0.53-0.75)	<0.001	0.63 (0.52-0.75)	<0.001
HGS-BH/WT	0.64 (0.55-0.75)	<0.001	0.70 (0.59-0.82)	<0.001	0.70 (0.59-0.83)	<0.001
HGS-BH/BMI	0.64 (0.55-0.76)	<0.001	0.71 (0.59-0.85)	<0.001	0.71 (0.59-0.86)	0.001
HGS-BH/WC	0.61 (0.52-0.72)	<0.001	0.66 (0.55-0.80)	<0.001	0.67 (0.54-0.81)	<0.001
HGS-BH/WHtR	0.62 (0.53-0.73)	<0.001	0.67 (0.55-0.82)	<0.001	0.67 (0.55-0.83)	<0.001

OR and p values were obtained from the crude and adjusted analyses using complex sample binary logistic regression. Odds ratios were estimated with 95% confidence intervals.

Model 1. Adjusted for age.

Model 2. Adjusted for age, residential area, education, employment status, household income, alcohol consumption, smoking status, walking exercise and menopause.

Abbreviations: HGS: handgrip strength, DH: dominant hand, BH: both hands, HGS-DH: maximum handgrip strength of the dominant hand, HGS-BH: maximum handgrip strength in both hands, HT: height, WT: weight, BMI: body mass index, WC: waist circumference, WHtR: waist-to-height ratio, OR: odds ratio, CI: confidence interval.

**Table 6:** Associations of OR-arthritis with absolute HGS and relative HGS indices in women.

Variables	Crude		Model 1		Model 2	
	OR (95% CI)	p value	Adj. OR (95% CI)	Adj. p value	Adj. OR (95% CI)	Adj. p value
Anthropometrics						
Height	0.71 (0.61-0.84)	<0.001	1.11 (0.93-1.34)	0.254	1.17 (0.96-1.43)	0.116
Weight	1.03 (0.87-1.22)	0.737	1.27 (1.06-1.52)	0.009	1.23 (1.02-1.48)	0.028
BMI	1.26 (1.06-1.49)	0.009	1.24 (1.03-1.49)	0.021	1.18 (0.97-1.43)	0.094
WC	1.52 (1.28-1.80)	<0.001	1.32 (1.10-1.59)	0.003	1.25 (1.04-1.51)	0.017
WHtR	1.65 (1.38-1.95)	<0.001	1.28 (1.06-1.55)	0.010	1.21 (0.99-1.47)	0.062
Absolute HGS						
HGS-DH	0.62 (0.52-0.73)	<0.001	0.94 (0.75-1.16)	0.548	0.93 (0.74-1.16)	0.530
HGS-BH	0.58 (0.49-0.69)	<0.001	0.88 (0.71-1.09)	0.244	0.87 (0.70-1.09)	0.232
Relative HGS						
HGS-DH/HT	0.64 (0.54-0.76)	<0.001	0.92 (0.75-1.13)	0.440	0.91 (0.74-1.12)	0.380
HGS-DH/WT	0.61 (0.51-0.73)	<0.001	0.81 (0.68-0.97)	0.023	0.82 (0.68-0.99)	0.035
HGS-DH/BMI	0.58 (0.48-0.70)	<0.001	0.83 (0.68-1.02)	0.078	0.85 (0.69-1.05)	0.141
HGS-DH/WC	0.54 (0.45-0.64)	<0.001	0.79 (0.64-0.98)	0.028	0.81 (0.65-1.00)	0.054
HGS-DH/WHtR	0.53 (0.44-0.63)	<0.001	0.80 (0.64-1.00)	0.051	0.83 (0.65-1.04)	0.106
HGS-BH/HT	0.60 (0.51-0.71)	<0.001	0.87 (0.71-1.06)	0.168	0.85 (0.70-1.05)	0.132
HGS-BH/WT	0.57 (0.48-0.69)	<0.001	0.76 (0.64-0.92)	0.004	0.77 (0.64-0.93)	0.007
HGS-BH/BMI	0.55 (0.45-0.66)	<0.001	0.78 (0.63-0.97)	0.022	0.80 (0.65-1.00)	0.051
HGS-BH/WC	0.50 (0.42-0.60)	<0.001	0.74 (0.60-0.91)	0.004	0.75 (0.61-0.93)	0.009
HGS-BH/WHtR	0.50 (0.42-0.59)	<0.001	0.75 (0.60-0.93)	0.010	0.77 (0.61-0.97)	0.027

OR and p values were obtained from the crude and adjusted analyses using complex sample binary logistic regression. Odds ratios were estimated with 95% confidence intervals.

Model 1. Adjusted for age.

Model 2. Adjusted for age, residential area, education, employment status, household income, alcohol consumption, smoking status, walking exercise and menopause.

Abbreviations: OR-arthritis: patients with both osteoarthritis and rheumatoid arthritis; HGS: handgrip strength; DH: dominant hand; BH: both hands; HGS-DH: maximum handgrip strength of the dominant hand; HGS-BH: maximum handgrip strength in both hands; HT: height; WT: weight; BMI: body mass index; WC: waist circumference; WHtR: waist-to-height ratio; OR: odds ratio; CI: confidence interval.

## Discussion

In this study, we demonstrated that the best indices among all indices from anthropometry and absolute and relative HGS indices differed according to OA, RA, and OR-arthritis status and sex. Specifically, in men, there were large differences in anthropometry (WHtR) and relative HGS (HGS-BH/WHtR) between patients with OA and nonarthritic individuals and large differences in relative HGS (HGS-DH/WC and HGS-DH/WHtR) between patients with RA and nonarthritic individuals. In women, there were differences in anthropometry (BMI) between patients with OA and nonarthritic individuals. Additionally, strong associations with RA were detected for absolute HGS (HGS-BH) and relative HGS (HGS-DH/HT), and strong associations with OR-arthritis were detected for relative HGS (HGS-BH/WC and HGS-BH/WT). Furthermore, the magnitude of associations between RA and HGS was greater than that between OA and HGS in both sexes.

To date, many studies have suggested that HGS is associated with OA [11–17] or RA [18–20,31–33] in several countries. However, no studies have examined the association between RA and relative HGS, and only a few studies have used HGS/BMI as a relative HGS index for the association between OA and HGS [16]. Regarding the association between RA and HGS, Björk et al. [32] investigated indicators of functional activity limitation in Swedish men and women with RA and reported that HGS significantly differed between RA patients and control participants in both sexes, and HGS was a strong indicator of health in both cross-sectional and longitudinal analyses. Žura et al. [18] examined the association between HGS and RA and assessed sex-specific differences in HGS between male and female patients with RA and nonarthritic subjects. They reported that both men and women with RA had weaker HGS than nonarthritic subjects, and men with RA had weaker HGS than women with RA compared to nonarthritic subjects. Additionally, Da Silva et al. [19] assessed differences in HGS between an RA patient group and a control group matched by age, sex, and BMI in Brazil. They argued that the HGS of RA patients was lower than that of control participants and that decreased HGS was a powerful predictor of functional disability in patients with RA. Sobue et al. [31] investigated the association between HGS and frailty in female patients with RA in Japan and demonstrated that HGS is associated with frailty when patients with RA have no joint symptoms. Kabul et al. [20] examined upper-extremity kinematics in women with RA and compared HGS and elbow flexion between patients and healthy subjects and reported that patients with RA had lower HGS and more elbow flexion than healthy subjects. In Korea, Lee et al. [33] argued that increased HGS was significantly related to a low prevalence of RA in older adults.

Regarding the association between OA and HGS, Zhang et al. [11] estimated the prevalence of hand OA and examined its impact on HGS using data provided by the Framingham Study and reported that the maximal HGS of patients with hand OA was 10% lower than that of nonarthritic subjects. Duruöz et al. [12] studied the clinical and functional characteristics of hand OA in the Turkish population and suggested that patients with hand OA had weaker HGS and functional disability than did the healthy population. Additionally, Chen et al. [13] examined the associations of OA and sarcopenia with HGS using cross-sectional analysis and Mendelian randomization in a US population. They argued that HGS was significantly associated with the risk of OA and that HGS had a significant role in the development of OA. Hochberg et al. [14] studied the metabolic and physiologic features associated with OA in Caucasian men in the US and reported that increased levels of hand OA were related to lower HGS. Dominick et al. [15] examined the association between radiographic indices and HGS among patients with hand OA in the US and argued that HGS was strongly associated with OA in carpometacarpal joints and that pinch strength was strongly related to OA in metacarpophalangeal joints. In Korea, Kim et al. [16] investigated the associations between relative HGS (HGS/BMI) and chronic cardiometabolic and musculoskeletal diseases and reported that low relative HGS was significantly associated with OA in both sexes. Wen et al. [17] examined the association between maximum HGS and radiographic OA in patients without hand joint pain and reported that low HGS was negatively associated with hand and knee OA in both elderly men and women. Our findings are consistent with the results of previous studies indicating that patients with RA have lower HGS than nonarthritic subjects or control participants [18–20] and suggesting that subjects with OA have lower HGS than healthy subjects [11–17]. Additionally, our results agree with the results of previous studies suggesting that the prevalence of OA is greater in women than in men [11,12,34] and that RA is more frequent in women than in men [6].

The biological and pathophysiological mechanisms underlying the association between HGS and OA or RA are still unclear, but several mechanisms may explain this association. OA is characterized by a complex mechanism of joint dysfunction and chronic inflammation [35], such as meniscal injury, degenerative changes in cartilage, joint space proliferation, and synovial inflammation [36,37]. Regarding possible mechanisms underlying the association between OA and HGS, several studies have suggested that a low level of inflammation is related to synovitis in OA [38–40], although generally, OA and RA are classified as noninflammatory diseases or inflammatory diseases [38]. In other research, a reduction in HGS was shown to be influenced by C-reactive protein (CRP) and high-sensitivity CRP (Hs-CRP) [16,41], and the Hs-CRP levels in the OA group were significantly greater than those in the control group and were associated with a decrease in muscle strength [42]. Additionally, an increased erythrocyte sedimentation rate (ESR) or CRP level was associated with decreased muscle strength in OA patients [38], and OA was strongly associated with decreased HGS [16,38]. On the other hand, insulin resistance indices such as homeostatic model assessment for insulin resistance (HOMA-IR), glucose, and insulin were influenced by HGS in both sexes, and metabolic risk factors such as total cholesterol and high-density lipoprotein-cholesterol in men and systolic blood pressure, DBP, low-density lipoprotein-cholesterol, and triglycerides in women were associated with HGS [16]. A recent Mendelian randomization study revealed a causal effect of OA on appendicular lean mass (ALM) and HGS and argued that ALM and HGS are causally related to OA [43]. Among the possible mechanisms underlying the association between RA and HGS, increased inflammation is associated with decreased HGS [44,45], and RA induces decreased HGS and muscle strength [25]. High levels of inflammatory markers of specific cytokines, such as CRP, interleukin-6 (IL-6), and IL-1RA, are related to worse HGS [45–47] and are negatively associated with low muscle strength in older individuals [45,46]. The overproduction of tumor necrosis factor α (TNF-α) and IL-1 leads to pathophysiological processes in RA [48]. For example, TNF-α and IL-6 lead to a decrease in skeletal muscle strength and are strongly associated with the development of autoimmune diseases [49]. On the other hand, decreased HGS was associated with joint inflammation, such as synovitis and tenosynovitis, in early RA [44]. Additionally, HGS was independently related to biological and functional factors. For example, patients with a lower HGS had lower hemoglobin and albumin levels and shorter survival [50]. A recent study reported the potential causality of RA and HGS, in which RA was causally positively associated with low HGS [49], but further studies are needed to reveal the exact mechanisms between HGS and RA/OA.

The present study had several limitations. First, the study cannot define the cause‒effect relationship between HGS and RA or OA because of the cross-sectional design of this study. Second, the KNHANES used in the present study supports doctor-diagnosed RA and OA and self-reported current RA and OA symptoms using questionnaires, rather than medical records or radiographic evidence. Therefore, although face-to-face health interviews were conducted by well-trained staff or experts in accordance with standardized guidelines to reduce recall bias, the findings of this study should be interpreted with caution. Finally, this study has additional limitations, including the absence of inflammatory cytokine–related analyses, insufficient stratification of smoking exposure by the number of cigarettes smoked per day, and the lack of detailed information on patients’ medications. Despite these limitations, the results of the present study were robust because of the use of a nationally representative sample of the Korean population using up-to-date KNHANES data with a very large sample.

In conclusion, low HGS is a potential index for predicting muscular strength and joint function and is a simple and cost-effective index related to various diseases and mortality. In this study, we examined the associations of OA, RA, and OR-arthritis with absolute and relative HGS indices in a Korean population and demonstrated that the best index among the anthropometric and absolute- and relative-HGS indices differed according to disease and sex. To our knowledge, this is the first study to report the associations of OA, RA, and OR-arthritis with various anthropometric indices, relative HGS, and absolute HGS indices.
